# IMpleMenting Effective infection prevention and control in ReSidential aged carE (IMMERSE): protocol for a multi-level mixed methods implementation study

**DOI:** 10.1186/s12877-023-03766-9

**Published:** 2023-02-23

**Authors:** Joanne Tropea, Sanne Peters, Jill J. Francis, Noleen Bennett, Deirdre Fetherstonhaugh, Kirsty Buising, Lyn-li Lim, Caroline Marshall, Madelaine Flynn, Michael Murray, Paul Yates, Craig Aboltins, Douglas Johnson, Jason Kwong, Karrie Long, Judy McCahon, Wen K. Lim

**Affiliations:** 1 Department of Aged Care, Royal Melbourne Hospital, Level 8 CRM, 300 Grattan Street, Parkville, VIC 3050 Australia; 2 Department of Medicine – Royal Melbourne Hospital, University of Melbourne, Parkville, VIC 3010 Australia; 3 School of Health Sciences, University of Melbourne, Parkville, VIC 3010 Australia; 4 Department of Public Health and Primary Care, University of Leuven, KU Leuven, Louvain, Belgium; 5 Department of Health Services Research, Peter MacCallum Cancer Centre, Melbourne, VIC 3000 Australia; 6 Department of Oncology, Sir Peter MacCallum, University of Melbourne, Parkville, VIC 3010 Australia; 7 Ottawa Hospital Research Institute – General Campus, Centre for Implementation Research, 501 Smyth Road, Ottawa, ON K1H 8L6 Canada; 8 Victorian Healthcare Associated Infection Surveillance System (VICNISS) Coordinating Centre and Department of Infectious Diseases, University of Melbourne at the Peter Doherty Institute for Infection and Immunity, Melbourne VIC 3000, Australia; 9 Department of Infectious Diseases, National Centre for Antimicrobial Stewardship, University of Melbourne, Melbourne, VIC 3000 Australia; 10 Department of Nursing, School of Health Sciences, University of Melbourne, Parkville, VIC 3010 Australia; 11 Australian Centre for Evidence Based Aged Care (ACEBAC), La Trobe University, Bundoora, VIC 3086 Australia; 12 Victorian Infectious Diseases Service, Royal Melbourne Hospital, Parkville, VIC 3050 Australia; 13 Department of Infectious Diseases, University of Melbourne at the Peter Doherty Institute for Infection and Immunity, Melbourne VIC 3000, Australia; 14 Infection Prevention and Surveillance Service, Royal Melbourne Hospital, Parkville, VIC 3050 Australia; 15 Director of Infection Prevention, Northern Health, Epping, VIC 3076 Australia; 16 Victorian Aged Care Response Centre, Australian Department of Health, Melbourne VIC 3000, Australia; 17 Department of Geriatric Medicine, Austin Health, Heidelberg, VIC 3084 Australia; 18 Department of Medicine – Austin Health, University of Melbourne, Heidelberg, VIC 3084 Australia; 19 Department of Infectious Diseases, Northern Health, Epping, Vic 3076 Australia; 20 Department of Medicine, Northern Clinical School, University of Melbourne, Epping VIC 3076, Australia; 21 Departments of General Medicine and Infectious Diseases, Royal Melbourne Hospital, Parkville VIC 3050, Australia; 22 Department of Infectious Diseases, Austin Health, Heidelberg VIC 3084, Australia; 23 Department of Microbiology & Immunology, University of Melbourne at the Peter Doherty Institute for Infection & Immunity, Melbourne VIC 3000, Australia; 24 Director Nursing Research Hub, Royal Melbourne Hospital, Parkville VIC 3050, Australia; 25 Consumer Representative of the IMMERSE Research Team, and Melbourne Academic Centre for Health, Parkville VIC 3050, Australia

**Keywords:** Infection prevention and control, Nursing homes, Residential aged care, Best practice, Implementation science, Organisational readiness, Behaviour change, Mixed methods

## Abstract

**Background:**

Older people living in residential aged care facilities are at high risk of acquiring infections such as influenza, gastroenteritis, and more recently COVID-19. These infections are a major cause of morbidity and mortality among this cohort. Quality infection prevention and control practice in residential aged care is therefore imperative. Although appointment of a dedicated infection prevention and control (IPC) lead in every Australian residential aged care facility is now mandated, all people working in this setting have a role to play in IPC. The COVID-19 pandemic revealed inadequacies in IPC in this sector and highlighted the need for interventions to improve implementation of best practice.

**Methods:**

Using mixed methods, this four-phase implementation study will use theory-informed approaches to: (1) assess residential aged care facilities’ readiness for IPC practice change, (2) explore current practice using scenario-based assessments, (3) investigate barriers to best practice IPC, and (4) determine and evaluate feasible and locally tailored solutions to overcome the identified barriers. IPC leads will be upskilled and supported to operationalise the selected solutions. Staff working in residential aged care facilities, residents and their families will be recruited for participation in surveys and semi-structured interviews. Data will be analysed and triangulated at each phase, with findings informing the subsequent phases. Stakeholder groups at each facility and the IMMERSE project’s Reference Group will contribute to the interpretation of findings at each phase of the project.

**Discussion:**

This multi-site study will comprehensively explore infection prevention and control practices in residential aged care. It will inform and support locally appropriate evidence-based strategies for enhancing infection prevention and control practice.

## Contributions to the literature

- This multi-level mixed-methods study will use a range of implementation science frameworks to investigate and inform infection prevention and control practice change in residential aged care.
- Findings will enhance our understanding of current practice, including organisational readiness, and barriers to implementation of best practice infection prevention and control in residential aged care.
- The research team will work in collaboration with infection prevention and control leads and other key stakeholders to determine and facilitate contextually tailored implementation strategies to overcome barriers to best practice infection prevention and control. This approach aims to optimise acceptability and sustainability of practice change.

## Background

Residential aged care facilities (RACFs) or nursing homes frequently experience outbreaks of common communicable infections such as viral respiratory tract infections and gastroenteritis which have significant consequences for residents. These outbreaks occur for several reasons. Residents live in close proximity to one another, share living areas and bathrooms, and are exposed to frequent close interactions with many different staff and visitors who might themselves carry infection [1]. Some residents exhibit behaviours that favour spread (wandering behaviours), and some do not have the capacity to following infection control interventions such as staying in their rooms, disinfecting hands, or practising respiratory etiquette. Residents are also generally old and frail, with multiple comorbidities, making them more vulnerable to significant morbidity and mortality from these infections. A 2018 national survey of Australian RACFs found 45% had experienced an influenza outbreak and 31% a gastroenteritis outbreak in the preceding 12 months; and 12% of these outbreaks were associated with deaths of residents [2]. The COVID-19 pandemic has further highlighted the vulnerability of this population; in Australia, the pandemic has disproportionately affected residents. As of 22 April 2022, a total of 3873 COVID-19 outbreaks had occurred in more than 2000 RACFs; over 31,000 residents contracted the virus and 2096 residents died with COVID-19 [3]. Deaths among residents made up 30% of all deaths associated with COVID-19 in Australia [4]; this figure was even higher prior to the rollout of COVID-19 vaccinations and the Omicron variant.

Staff caring for people in RACFs must therefore ensure effective infection prevention and control (IPC) practices are in place both to prevent and respond to infections. These practices include early recognition of infection and action to contain the source using appropriate cohorting or isolation strategies, personal protective equipment (PPE), and hand hygiene [5]. Effective IPC not only requires a workforce with IPC knowledge and skills, but needs systems, administrative and environmental controls which enable appropriate behaviours [6].

Investigations into COVID-19 outbreaks in Australian RACFs highlighted IPC challenges at multiple levels [7, 8]. Organisational and system level challenges that contributed to outbreaks were identified, including poor leadership and management skills, problems with human resources most notably severe staff shortages, difficulties with procurement of PPE and other supplies; problems with the physical layout of buildings, lack of space and inability to separate residents or staff workflows. Issues at the team and individual staff level were also reported, including suboptimal staff communication strategies, inadequate training of staff, and lack of access to clear information in a timely way. To provide further context, Table 1 below describes the Australian residential aged care sector and some of the workforce and resource challenges faced by the sector. It also describes the introduction of IPC leads that the Australian government mandated in response to the COVID-19 outbreaks in aged care.

**Table 1:** Residential Aged Care Sector: context of the current study

Australia’s residential aged care sector is complex, with over 800 residential aged care providers from the private (for-profit), not-for-profit, and government sectors operating over 2700 RACFs [9]. Workforce turnover in the sector is high: the 2020 Australian Workforce Census reported 29% annual direct care staff turnover and 37% turnover of registered nurses in residential aged care compared to the national average of 7.5%. [10, 11]
Compared to hospitals, RACFs have fewer IPC resources such as on-site clinical staff with IPC expertise, and many have less direct access to diagnostic and support services. Staff responsible for IPC usually have multiple other responsibilities and are not trained to the same level as IPC practitioners in hospitals [12, 13]. There is also wide variation in staffing levels and skills mix in general, as highlighted by the Royal Commission into Aged Care Quality and Safety (2021) which found over half the residents were living in facilities with unacceptable levels of staffing [14]. Studies have also shown lower levels of staffing in RACFs, in particular, low proportions of registered nurses, were associated with greater risk of COVID-19 outbreaks [15, 16]
**Aged Care IPC leads**: As part of the Australian Government’s response to COVID-19 outbreaks in residential aged care, as of 1 December 2020 every RACF is required to appoint an on-site nurse as the IPC lead. The IPC lead role is to ensure the RACF is optimally prepared to prevent and respond to infectious diseases [17]. IPC leads must complete a specialist training course, be employed by the facility, and report directly to the provider. They are required to observe, assess, and report on IPC practices, help develop procedures, and provide advice to improve IPC within the service [17]
Several challenges related to the Aged Care IPC lead program have been reported, including a lack of a clear role description, new processes that added burden to an already overburdened job, issues related to the IPC lead training such as having to complete the training in a short period of time (6-month course condensed into 3-months) [18]. Many IPC leads have been appointed from existing members of the nursing staff and they tend to have a much broader role in the facility

The IPC challenges described above are not unique to COVID-19 but apply to other infectious diseases. A rapid Cochrane review, undertaken at the beginning of the COVID-19 pandemic to inform COVID-19 management, explored organisational, environmental, and individual barriers and facilitators to healthcare workers’ adherence to IPC guidelines for respiratory infections [19]. They found organisational factors such as high workload, limited training, limited PPE; and environmental factors such as insufficient space to isolate patients, anterooms and bathroom facilities influenced their ability to adhere to IPC guidelines. Individual-level factors were described in terms of healthcare workers’ attitudes, knowledge, and beliefs. Perceived enablers included feeling supported by managers; seeing value in the guideline; being motivated by fear of infecting themselves, their family, and others; feeling responsible for their patients; and feeling their safety was valued by management. Most studies included in the review were conducted in hospitals; and the authors acknowledged the lack of research in RACFs [19].

To date, improvement in evidence-based practice in Australian RACFs has been largely driven at the system level by mandated requirements. These include government directives mandating staff influenza vaccinations [20]. Although many initiatives to improve IPC have been introduced, IPC implementation studies in aged care are lacking; and implementation strategies to improve IPC practice from other settings such as hospitals are often not transferrable to RACFs because of differences in the skill mix of the staff, complexity of the residents’ conditions, and the limited availability of IPC expertise and resources [21]. In addition, there is a poor understanding of how change processes can take place and how contextual factors might influence the effectiveness of implementation strategies [22]. What is needed is a clear evidence base to guide how IPC practices can most effectively be implemented in RACFs that take into account variation at both organisational and individual staff levels. Calls for action to improve IPC in RACFs have clearly been made, but such interventions need to be guided by evidence to optimise the likelihood of success.

The IMMERSE study aims to address these gaps in the evidence base. It is a mixed-methods, theory-informed, multi-level implementation project that will use an iterative approach and work in collaboration with IPC leads. In acknowledgement of the key role of contextual factors in supporting change, organisational readiness for IPC practice change will be investigated [23, 24]. A full range of behaviours required from various staff that contribute to effective IPC will be explored using a structured approach (the Actor, Action, Context, Target, Time framework [25]). Localised barriers to performing these behaviours will be investigated using the Theoretical Domains Framework (TDF) to identify priority domains which can be addressed by specific behaviour change techniques (BCTs) [26, 27].

Local customisation is key for IPC practices, and it is expected that a ‘one size fits all’, ‘top-down’ approach is unlikely to be successful. The IMMERSE research team will therefore work in close collaboration with the IPC leads and other key RACF staff to operationalise the selected BCTs in ways that are feasible and appropriate at each RACF. The project will also explore the acceptability of a community of practice for IPC leads to share learnings and resources, to support communication and networking, and provide up-to-date information [28, 29]. Similar community of practice models have been shown to improve healthcare provider knowledge, improve role certainty, provide social support that fosters change in provider behaviour and hence improve patient outcomes [30].

## Methods

### Study aims

The study aims are to: (i) assess organisational readiness for IPC practice change in participating RACFs; (ii) specify and prioritise component behaviours of good IPC practice including actions performed by a full range of RACF staff; (iii) identify barriers experienced and anticipated by a full range of RACF staff in performing the identified component behaviours of good IPC practice; (iv) determine feasible, locally relevant, and acceptable solutions to address the identified barriers; and (v) upskill the IPC leads to facilitate practice changes to improve and sustain IPC, including the potential for a community of practice to support IPC leads.

### Study design

**Fig. 1:**
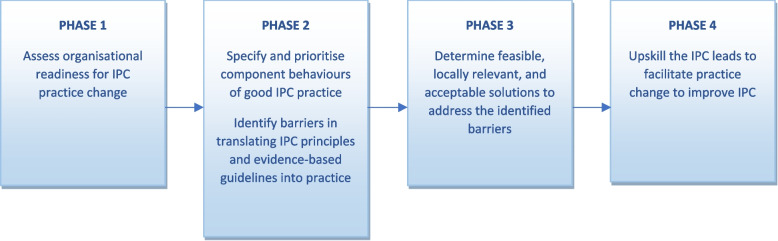
Study phases

This is a multilevel, mixed-methods implementation study, with four phases (Fig. 1). Stakeholder groups at each facility and the IMMERSE project’s Reference Group (carer advocacy, industry, and government representatives) will contribute to the interpretation of findings at each phase of the project. This study was approved by the Melbourne Health Human Research Ethics Committee (HREC/81901/MH-2022) for conduct of the study across all sites.

### Setting and inclusion criteria

RACFs in metropolitan Melbourne and regional Victoria are eligible to participate. Ten RACFs will be recruited, with representation of metropolitan and regional locations, small, medium, and large sized, private, public, and not-for-profit providers, and culturally and linguistically diverse RACFs. Purposive sampling will be used to ensure representation from a diverse range of RACFs. RACFs suggested by the IMMERSE investigators (who have extensive networks and depth knowledge of RACFs in the region) will be invited to participate. Information leaflets will be sent with an email invitation to RACF managers of potential RACFs, and online meetings will be held with members of the project team and the RACF manager and IPC lead to introduce the research team and present background information, benefits, and risks of being involved in the project and what would be involved. The IPC leads are crucial participants in the project, and only RACFs with IPC leads who agree to consent to participate will be included. Collaborative research agreements between the RACF providers and the IMMERSE lead investigator will be documented and signed.

### Participants

#### Staff

A full range of staff employed at the RACFs will be invited to participate in the study, including facility and clinical managers, IPC leads, nurses, personal carers, food services and cleaning staff, and other direct care and ancillary staff.

#### Residents and family visitors

Residents and family visitors who can understand English, and residents with the capacity to give informed consent will be invited to participate in the study. Family participants must be at least 18 years old

### Data collection and analysis

**Phase 1** will explore organisational readiness for change and existing IPC program components at the level of individual sites. There will be three data collection activities: (i) staff survey of organisational readiness for change; (ii) IPC lead and/or facility manager survey of local IPC program components; and (iii) follow-up interviews with staff, residents, and family visitors about their experiences of IPC program components.

#### Survey of organisational readiness for change

The staff survey of organisational readiness for change consists of the Organizational Readiness for Implementing Change (ORIC) [31], and items from the Organizational Readiness to Change Assessment (ORCA) Context Assessment scale [32]. The ORIC is a validated tool for measuring organisational readiness. It assesses change commitment (5 statements) and change efficacy (7 items). Each of the 12 items is scored using a 5-point Likert scale ranging from “Disagree” to “Agree”. The ORCA instrument consists of 77 items measured on a 5-point Likert scale to assess three primary scales—evidence, context, and facilitation. Items relevant to the project were selected from each subscale of the context scale, and the Likert scale was modified to be consistent with the ORIC scale. All staff will be invited to complete the organisational readiness for change survey. Participation in the survey will be voluntary and consent implied. This approach, as opposed to seeking prior consent, will facilitate greater uptake of the survey and increase likelihood of sufficient responses at each facility to be able to infer a valid organisational-level response.

#### Survey of IPC program components

IPC leads and facility managers will be asked to complete the survey of IPC program components. The survey has been developed for the purposes of this research; item development was informed by a review of the literature on core components of IPC programs in RACFs, international and national IPC guidelines, and input from the IMMERSE Research Team and Advisory Group. It includes items on IPC personnel, IPC policies and procedures, staff IPC training, resident and family engagement, staff and resident health and safety, surveillance, IPC program reporting and governance, and antimicrobial stewardship.

The surveys will be pilot tested at non-participating facilities using think-aloud interviews to assess whether items are clear and elicit expected responses [33]. They will be tested among staff from culturally and linguistically diverse backgrounds, and where required, changes will be made in response to their needs.

Surveys will be sent electronically via email or short message service (SMS) using Research Electronic Data Capture (REDCap) [34]. Hard copy versions will be sent upon request or at RACFs with low uptake. Research staff will enter hard copy survey responses manually into REDCap.

#### Follow-up interviews

Follow-up site visits will be conducted to verify the IPC program survey responses and collect further details on the IPC program and organisational readiness via semi-structured interviews with staff, residents, and family visitors. Individual or small group interviews will be conducted at each site with a purposive sample of eight multidisciplinary staff, one or two residents, and one family member. The researchers will use a “walk-and-talk” approach to verify locations of IPC-related procedures, staff training logs, equipment, and resources. The “walk-and-talk” approach will take place within the RACF, and it functions as a situated interview in which contextual triggers might enhance the participants’ descriptions and make the responses to the interview questions more concrete and locally relevant. The researchers will take notes during and after the interviews, and a summary of the interviews will be sent back to participants for verification. Interviews with family members and residents will collect information about their involvement and engagement in IPC at the RACF. Interviews with residents and family members will be audio-recorded and transcribed verbatim for analysis, and the researchers will take notes (for example, how and where the interview took place, interruptions, non-verbal cues). The researchers will also collect data on turnover of senior staff and management for the duration of the project.

#### Recruitment of residents and families for interviews

Residents and family carers from the RACFs will be invited to participate in face-to-face or phone interviews. IPC leads and other senior nursing staff will be asked to nominate residents who have capacity to consent to participate, and to provide the contact details of family carers who may be interested in participating. Input from family members of residents who do not have capacity will be sought, for example family members of residents with advanced dementia.

While on site visits, research staff will approach residents and family visitors in person to inform them about the project and invite them to participate. In the event of difficulty recruiting family carers for interviews, letters of invitation with the plain language statement will be sent by post or email to family carers and follow up phone calls made by the researchers. Interviews will then be scheduled with family members interested in participating.

Participation in interviews is voluntary. Those who agree to participate will be provided with plain language statement and written consent will be obtained. Verbal consent will be sought from family members who agree to participate in interviews by phone or web-conference. As a token of appreciation, the IPC leads and other staff who participate outside their work hours, and participating residents and family members will be offered a gift voucher as acknowledgement of the time taken to be interviewed.

#### Data analysis

Survey data will be exported from REDCap into statistical software for analysis, and descriptive statistics will be used to summarise survey item responses. Initial exploration of ORIC and ORCA survey scores will include inspecting overall RACF-level responses. If warranted, further exploration of differences between RACF group responses and healthcare worker groups will be examined using one-way analysis of variance (ANOVA) for parametric distributed or Kruskal–Wallis for non-parametric distributed data, with level of significance set at p level = 0.05.

Interview notes and transcripts will be coded and analysed by two researchers (JT, SP) using thematic analysis [35, 36]. The coding process will take an inductive approach, with themes and codes identified and derived from the data rather than working with pre-identified themes and codes. Initially, both researchers will code the interviews from the first RACF together to formulate a codebook. They will then code the second interview independently and will review the coding together and reach consensus through discussion, amending the codebook if required for further clarity. Coding of the remaining interviews will be completed by one researcher, with a small sample independently double coded to assess interrater reliability (Kappa; κ).

Analysis will proceed separately for each RACF. The QSR NVivo software program [37] will be used to assist with storage, coding and searching of data. Survey and interview data will be triangulated using structured methods, as described in Hopf and colleagues (2016) [38], to assess the extent to which the RACF is ready for innovation and has the appropriate IPC infrastructure in place.

#### Data reporting

A summary of the findings will be presented to participating facilities. Where areas for improvement are identified, evidence-based solutions will be utilised. Where there little or no evidence base, the researchers in collaboration with RACF staff will determine solutions and make recommendations to address them. These recommendations will leverage simple, less costly, and existing solutions (resources, networks and supports) and where more costly and time-consuming solutions are required, we will advocate for the development of specific resources and supports. RACFs will then be invited to participate in Phase 2 of the project.

**Phase 2** will investigate staff behaviours relating to current IPC practice and compare this to guideline recommendations to identify gaps in practice. This will be followed by identification of barriers to translating IPC guideline recommendations and principles into practice. This phase consists of three key activities: (i) scenario-based assessment of IPC practice; (ii) document analysis of key IPC procedures; and (ii) exploration of barriers using the TDF.

#### Scenario-based assessment

Staff will be guided through hypothetical sequential descriptions of two common IPC scenarios; one focussing on reactive and one on proactive IPC practice. These scenarios will be developed by the IMMERSE Research Team; and the preferred sequence of actions for each scenario will be mapped out using the Action, Actor, Context, Target, Time (AACTT) framework for specifying behaviour, including team-based behaviours [25].

For each scenario, staff will be asked to describe individual and team level behaviours, including how they make decisions and what actions they would do, in response to sequential scenario descriptions. Application of the AACTT framework during this step will be used to help identify which individuals (Actors) at which levels of an organisational hierarchy need to do something differently to perform the specified evidence-based IPC practice (action) for certain residents (target) at an appropriate time and in a specific setting (context). The interview topic guide will be based on the AACTT-specified behaviours. Interviews will be audio-recorded and transcribed verbatim.

Responses to the scenario-based assessment will be summarised according to the domains of the AACTT framework. The matrix generated by participants will be compared to the ideal sequence matrix developed by the IMMERSE research team (based on guideline documents; described below). The findings will highlight areas of good IPC practice and areas of suboptimal IPC practice and will identify behaviours that could be improved.

#### Document analysis

Document analyses of key IPC policies and procedures from participating RACFs will be conducted. The IPC procedures will be coded into the domains of the AACTT framework, will be rated for specificity and summarised in matrix form. The matrix generated from the document analysis will then be compared to the national guideline matrix developed by the IMMERSE research team.

The findings from the scenario-based assessment and document analyses will be presented to staff at each of the participating RACFs; and priority areas for improvement will be agreed in collaboration with IPC leads and other key stakeholders, using a consensus approach [39].

#### Exploration of barriers

The findings from the above activities will then be used to guide the follow-up staff interviews. This allows more focused investigations into the barriers and drivers of Actions for each Actor group and will inform our approach to measuring the success of the implementation strategies in terms of behavioural outcomes (i.e., do the specified Actors engage in the specified Actions at the appropriate Times and Places?). We will likely focus on three or four behaviours or actions that need to change from the scenario-based assessment to explore the barriers in translating best practice IPC recommendations into practice. The ‘Actors’ identified in the previous activity—those staff who need to do something differently, will be invited to participate in semi-structured interviews (one-on-one or small groups). The interview topic guide will be developed based on the TDF domains using published methods [40]. Interviews will be audio-recorded and transcribed verbatim. Transcripts will be imported into QSR NVivo for analysis.

Interview transcripts will be coded and analysed by two researchers (JT, SP). Initially, both researchers will code the first interview together to formulate the coding strategy, using the TDF domains as a guide. They will then code the second interview independently and will review the coding together and reach consensus through discussion. Coding of the remaining interviews will be completed independently, with a small sample double coded to assess interrater reliability (Kappa; κ). Data will initially be coded deductively into theoretical domains, then inductively to identify specific barriers and enablers within each domain according to TDF guidance [40].

Analysis will proceed separately for each site (to identify site-specific barriers) and for each professional group. The ‘COnsolidated criteria for REporting Qualitative research’ (COREQ) checklist will be used to enhance the reporting of our research [41]. Findings will be summarised and used to inform Phase 3 activities.

**Phase 3** will map BCTs to address the identified barriers to best practice IPC and determine feasible and acceptable solutions.

Systematic mapping methods will be used to map BCTs that address the individual- and team-level barriers to the priority behaviours identified in Phase 2. The matrix developed by Michie et al. will be used, which links a taxonomy of BCTs to TDF domains and indicates which BCTs are likely to be effective in changing that particular domain with a view to supporting behaviour change [26, 42].

The findings will then be presented to the IMMERSE team and other key stakeholders to establish potential modes of delivery in terms of feasibility and appropriateness. At each RACF, we will work in collaboration with IPC leads and other key staff to select the most appropriate BCTs (solutions) and work with them to operationalise the techniques in a way that has good fit within the context. The aim of these sessions will be to select feasible and fit-for-purpose intervention strategies and intervention components. Criteria for success will be agreed by stakeholders and are likely to include the measures of staff satisfaction, staff turnover, IPC lead self-efficacy, organisational readiness.

**Phase 4** will apply evidence-based techniques with a view to upskilling the IPC leads to facilitate IPC practice change and explore the potential of an IPC lead community of practice.

The research team will work directly with IPC leads to assess acceptability of selected BCTs or solutions. We will use evidence-based techniques to upskill IPC leads to enable them to deliver the selected BCTs to their colleagues, for example goal setting in relation to IPC practices together with monitoring and feedback, building self-efficacy, building a safety culture to improve collective practice, and embedding reminders that fit clinical workflow. Training sessions for upskilling will be conducted and may involve simulation-based training, peer-to-peer mentoring, and feedback in the context of positive social interactions. Sharing of local experiences and resources will be facilitated by a member of the research team with behaviour change expertise so IPC leads can learn from one another, including exploration of a community of practice for IPC leads.

## Discussion

This study will address a much-needed area of research. Prior to COVID-19, few published studies had investigated IPC practice in RACFs. Barriers to implementation of evidence-based IPC in RACFs have been explored in relation to hand hygiene, influenza management and antimicrobial stewardship [43–45]. However, theory- and evidence-informed implementation studies to overcome these barriers are lacking. Training, upskilling, and educating RACF staff have been the focus of most provider and government strategies to improve IPC practice. These strategies are important and can address gaps in knowledge and skills, however they do not address other factors that can impact on IPC practice, such as environmental context and resources, reinforcement, motivation, and social influences.

The IMMERSE study seeks to address these gaps in research by comprehensively exploring IPC practice in RACFs and using multiple frameworks to inform and support IPC practice change. The study is novel in design, being, to our knowledge, the first to assess organisational readiness to change and how it impacts on supporting IPC practice change; the first to apply the AACTT framework to explore staff behaviours in response to common IPC scenarios; and the first to investigate barriers to best IPC practice using the TDF, and to then map and deliver feasible BCTs to overcome these barriers. The IMMERSE researchers will work directly with IPC leads to operationalise the BCTs. Working in collaboration with IPC leads and other key stakeholders will optimise acceptability and sustainability of solutions.

This is an opportunity to transform the care provided to older people living in RACFs by improving IPC through identifying and addressing organisation-, team-, and individual-level barriers to effective IPC practice, and supporting IPC leads to facilitate IPC practice change.

## Data Availability

The datasets used and/or analysed during the current study will be available from the corresponding author on reasonable request.

## Abbreviations

AACTT: Action, Actor, Context, Target, Time
ANOVA: Analysis of variance
BCT: Behaviour change techniques
COREQ: Consolidated criteria for reporting qualitative research
IMMERSE: IMpleMenting Effective infection prevention and control in ReSidential aged carE
IPC: Infection prevention and control
MRFF: Medical Research Future Fund
ORCA: Organizational Readiness to Change Assessment
ORIC: Organizational Readiness for Implementing Change
PPE: Personal protective equipment
RACFs: Residential aged care facilities
REDCap: Research Electronic Data Capture
SMS: Short message service
TDF: Theoretical Domains Framework
